# Production of Extracellular Laccase from *Bacillus subtilis* MTCC 2414 Using Agroresidues as a Potential Substrate

**DOI:** 10.1155/2015/765190

**Published:** 2015-09-14

**Authors:** Narayanan P. Muthukumarasamy, Beenie Jackson, Antony Joseph Raj, Murugan Sevanan

**Affiliations:** Department of Biotechnology, School of Biotechnology and Health Sciences, Karunya University, Coimbatore 641114, India

## Abstract

Laccases are the model enzymes for multicopper oxidases and participate in several applications such as bioremediation, biopulping, textile, and food industries. Laccase producing bacterium, *Bacillus subtilis* MTCC 2414, was subjected to optimization by conventional techniques and was partially purified using ammonium salt precipitation method. The agroresidue substrates used for higher yield of laccase were rice bran and wheat bran. Maximum production was achieved at temperature 30°C (270 ± 2.78 U/mL), pH 7.0 (345 ± 3.14 U/mL), and 96 h (267 ± 2.64 U/mL) of incubation. The carbon and nitrogen sources resulted in high enzyme yield at 3% sucrose (275 ± 3.11 U/mL) and 3% peptone (352.2 ± 4.32 U/mL) for rice bran and 3% sucrose (247.4 ± 3.51 U/mL) and 3% peptone (328 ± 3.33 U/mL) for wheat bran, respectively. The molecular weights of partially purified laccase were 52 kDa for rice bran and 55 kDa for wheat bran. The laccase exhibited optimal activity at 70°C (260.3 ± 6.15 U/mL), pH 9.0 (266 ± 4.02 U/mL), and metal ion CuSO_4_ (141.4 ± 6.64) was found to increase the production. This is the first report that delivers the higher yield of laccase produced from *B. subtilis* MTCC 2414 using agroresidues as a potential substrate.

## 1. Introduction

Laccases (p-benzenediol: oxygen oxidoreductase EC 1.10.3.2) are the members of multicopper protein family, belonging to the group of blue-copper proteins, and are widely distributed among plants, fungi, and bacteria [1]. Due to broad substrate specificity and ability to oxidize wide range of phenols and polyphenols, laccases have received much attention from researchers in the last decade. They also play a vital role in detoxification of textile effluents and bioremediation applications [2].

The existence of laccase in bacteria was reported in* B. halodurans*,* B. subtilis* SF,* Bacillus sp*. HR03,* Azospirillum lipoferum*,* P. desmolyticum* NCIM 2112,* B. pumilus*,* B. subtilis* WP1, and* P. putida,* respectively [3]. Most bacterial laccases are highly thermotolerant and maintain their activity in neutral to alkaline conditions [4], whereas fungal laccases usually drop their activities rapidly at high temperatures and pH. It is well known fact that the majority of agroindustrial wastes are lignocellulosic in nature. The production of laccase from these agroindustrial wastes is of great significance owing to its reuse of industrial waste which reduces the problems arising during disposal and also residues can be utilized for various industrial applications [5]. The selection of suitable natural substrate is greatly influenced by high lignin contents [6]. Moreover, these substrates provide a favourable natural habitat for the secretion of lignocellulolytic enzymes in larger amounts [7, 8]. Therefore, in the present study, wheat bran, rice bran, saw dust, and banana peels have been selected for the production of laccase.

Production of laccases is affected by many factors such as medium composition, time, pH, temperature, and carbon and nitrogen ratio [9]. The optimal temperature and pH are mainly dependent on the substrate for high yield of laccase [10]. Partial purification of laccase enzyme was carried out using ammonium sulphate precipitation method followed by dialysis [11]. The stability of laccase enzymes at different pH, temperature, and metal ions effect has to be demonstrated for its betterment in industrial applications. The present work aimed to ascertain the reliability of natural substrates such as rice bran and wheat bran for the enhanced production of laccase enzyme using* B. subtilis* MTCC 2414 under solid state fermentation. Moreover, optimization, partial purification, and characterization of laccase were also carried out in order to study their potential towards wide applications in biotechnology.

## 2. Methods

### 2.1. Microorganism and Substrate

*B. subtilis* MTCC 2414 was procured from Microbial Type Culture Collection (MTCC) center, Chandigarh, India. The strain has been tested for laccase producing ability through plate test method [12]. The synthetic substrate guaiacol (Hi-Media Laboratory, Mumbai) was employed for laccase activity determination.

### 2.2. Selection of Natural Substrates

Different natural substrates were screened based on their lignin contents to identify their suitability for enhanced laccase production using* B. subtilis* MTCC 2414. Natural substrates such as saw dust, banana peel, rice bran, and wheat bran were collected from the local markets in and around Coimbatore. The substrates were then individually washed 2-3 times with distilled water and boiled for 15 min. The water was then decanted and the substrates were dried in an oven at 60°C and powdered. The powder was sieved using a 40-micron mesh and stored.

#### 2.2.1. Substrate Analysis

The chemical compositions such as cellulose, hemicellulose, lignin (acid detergent fiber (ADF), acid detergent lignin (ADL), and neutral detergent fiber (NDF)), and ash content for different natural substrates were determined according to Norman and Jenkins [13] and O'Dwyer, [14]. Also, the effects of substrate concentrations (3–5% w/v) on laccase production were evaluated.

### 2.3. Production of Enzyme under SSF Condition

Exactly, 2 g of wheat bran and rice bran was taken in different flasks and moistened with 100 mL of Mineral Basal Salt Solution (MBSS) containing (g/L): peptone 3, dextrose 10, K_2_HPO_4_ 0.4, KH_2_PO_4_ 0.6, MnSO_4_ 0.5, FeSO_4_ 0.0005, and ZnSO_4_ 0.01. The initial moisture level in the medium was 20% (w/v). The flasks were sterilized, cooled to room temperature, and inoculated with 3.5 × 10^6^ CFU of* B. subtilis* MTCC 2414 and incubated for 24 h. After incubation, flasks were mixed thoroughly with 50 mM glycine-NaOH buffer (pH 9.0) under shaking conditions and centrifuged at 10000 rpm for 10 min at 4°C.

### 2.4. Optimization of Process Parameters

The various process parameters that influence the enzyme production during SSF were optimized over a wide range. The strategy adopted for standardization of process parameters was to evaluate the effect of an individual parameter and to incorporate it at standardized level before standardizing the next parameter.

### 2.5. Effect of Incubation Period on Enzyme Activity

Incubation period is an important factor for the production of enzyme. Fifty mL of MBSS and 2 g of wheat bran and rice bran were taken in 250 mL Erlenmeyer flasks. The flasks were sterilized, cooled to room temperature, and inoculated with 3.5 × 10^6^ CFU of* B. subtilis* MTCC 2414 and incubated at different time intervals, namely, 0, 24, 48, 72, 96, 120, and 144 h, respectively at room temperature (28 ± 2°C). The contents of the flasks were centrifuged at 10000 rpm for 10 min at 4°C and the supernatant was used to assay the enzyme activity at 420 nm.

### 2.6. Effect of Temperature at Optimized Incubation Period on Enzyme Activity

Environmental temperature is a factor to which the biomass is an inescapable subject, since cell temperature must become equal to the temperature of culture medium. Temperature affects the rate of cell reaction, nature of metabolism, nutritional requirement, and the biomass concentration. Fifty mL of MBSS and 2 g of wheat bran and rice bran were taken in 250 mL Erlenmeyer flasks. The flasks were sterilized, cooled to room temperature, and inoculated with 3.5 × 10^6^ CFU of* B. subtilis* MTCC 2414 and incubated at different temperatures (25, 30, 35, 40, 45, and 50°C) under optimum incubation period of 96 h. The contents of the flasks were centrifuged at 10000 rpm for 10 min at 4°C and the supernatant was used to assay the enzyme activity at 420 nm.

### 2.7. Effect of pH at Optimized Incubation Period and Temperature on Enzyme Activity

The influence of hydrogen ions on biological activities is related to their hydrogen ion concentration on enzyme activity. Fifty mL of MBSS and 2 g of wheat bran and rice bran were taken in 250 mL Erlenmeyer flasks and pH was adjusted in each of the flasks from 4.5, 5.0, 5.5, 6.0, 6.5, 7.0, 7.5, and 8.0, respectively, by addition of 1 N HCl and 1 N NaOH. The flasks were sterilized, cooled to room temperature, and inoculated with 3.5 × 10^6^ CFU of* B. subtilis* MTCC 2414 and incubated at optimized incubation period (96 h) and temperatures (30°C for rice bran and 40°C for wheat bran). The contents of the flasks were centrifuged at 10000 rpm for 10 min at 4°C and the supernatant was used to assay the enzyme activity at 420 nm.

### 2.8. Effect of Carbon Sources on Enzyme Activity

The nature and amount of carbon sources in the culture medium are important for the growth and production of extracellular laccase by bacteria. The production medium was enriched with varying concentrations (1–5%) of carbon sources, namely, glucose, maltose, sucrose, starch, and carboxymethylcellulose (CMC), in 250 mL Erlenmeyer flasks containing 50 mL of MBSS medium with 2 g of wheat bran and rice bran. The flasks were sterilized, cooled to room temperature, and inoculated with 3.5 × 10^6^ CFU of* B. subtilis* MTCC 2414 and incubated at optimized incubation period (96 h), temperatures (30°C for rice bran and 40°C for wheat bran), and pH (7.0 for both rice and wheat bran). The contents of the flasks were centrifuged at 10000 rpm for 10 min at 4°C and the supernatant was used to assay the enzyme activity at 420 nm.

### 2.9. Effect of Nitrogen Sources on Enzyme Activity

Metabolic concentration and the growth of bacteria are strongly influenced by medium composition, such as nitrogen sources. The production medium was enriched with varying concentrations (1–5%) of inorganic and organic nitrogen sources, namely, ammonium sulphate ((NH_4_)_2_SO_4_), sodium nitrate (NaNO_3_), potassium nitrate (KNO_3_), peptone, and beef extract in separate Erlenmeyer flasks containing 50 mL of MBSS medium with 2 g of wheat bran and rice bran. The flasks were sterilized, cooled to room temperature, and inoculated with 3.5 × 10^6^ CFU of* B. subtilis* MTCC 2414 and incubated at optimized incubation period (96 h), temperature(s) (30°C for rice bran and 40°C for wheat bran), and pH (7.0 for both rice and wheat bran). The contents of the flasks were centrifuged at 10000 rpm for 10 min at 4°C and the supernatant was used to assay the enzyme activity at 420 nm.

### 2.10. Production of Enzyme at Optimum Influencing Conditions

Erlenmeyer flasks (250 mL) containing 50 mL MBSS and 2 g of wheat bran and rice bran along with the optimized carbon and nitrogen sources such as 3% sucrose and 3% peptone were sterilized, cooled to room temperature, inoculated with 3.5 × 10^6^ CFU of* B. subtilis* MTCC 2414, and incubated at optimized incubation period (96 h), temperatures (30°C for rice bran and 40°C for wheat bran), and pH (7.0 for both rice and wheat bran). The contents of the flasks were centrifuged at 10000 rpm for 10 min at 4°C and the supernatant was used to assay the enzyme activity at 420 nm.

### 2.11. Determination of Laccase Activity

Laccase activity was measured by monitoring the oxidation of 1 mM guaiacol (Hi-Media, Mumbai, India) buffered with 0.2 M sodium phosphate buffer (pH 4.5) at 420 nm for 1 min. The reaction mixture (900 *µ*L) contained 300 *µ*L of 1 mM guaiacol, culture filtrate, and 0.2 M sodium acetate buffer (pH 4.5). One unit of enzyme activity was defined as the amount of enzyme that oxidized 1 *µ*mol of guaiacol per minute. The enzyme activity was expressed in U/mL [15]. The protein concentration was measured by Lowry et al.'s method using bovine serum albumin (BSA) as a standard [16].

### 2.12. Partial Purification of Laccase

The supernatant obtained after centrifugation was concentrated by fractioned precipitation of 80% ammonium sulphate saturation. Flasks were resuspended in 100 mL of 50 mM glycine-NaOH buffer (pH 9.0) and centrifuged at 10000 rpm for 10 min. After centrifugation, the sample having maximum enzyme activity was extensively dialyzed against phosphate buffer (50 mM, pH 8.0) and used for further studies. The experiments were carried out at room temperature (28 ± 2°C).

### 2.13. Determination of Molecular Weight

The molecular weight of laccase was determined using sodium dodecyl sulfate polyacrylamide gel electrophoresis (SDS-PAGE) using 12% polyacrylamide gel and the bands were visualized with Coomassie Brilliant Blue R [17].

### 2.14. Enzyme Characterization

#### 2.14.1. Temperature Profile

The thermos stability of partially purified laccase was analysed at different temperatures, namely, 50, 60, 70, 80, 90, and 100°C. At each temperature, 40 *µ*L of enzyme along with 2 mM guaiacol was incubated for 10 min. The contents of the flasks were centrifuged at 10000 rpm for 10 min at 4°C and the supernatant was used to assay the enzyme activity at 420 nm.

#### 2.14.2. pH Profile

Three different buffers (50 mM) were used for pH ranging from 4 to 11. Acetate buffer was used for pH 4 to 6. Phosphate buffer was used for pH 6 to 8 and glycine-NaOH buffer was used for pH 8 to 11. Forty *µ*L of enzyme and 2 mM guaiacol and respective buffers were incubated for 3 h at optimized temperature of 70°C. The contents of the flasks were centrifuged at 10000 rpm for 10 min at 4°C and the supernatant was used to assay the enzyme activity at 420 nm.

#### 2.14.3. Effect of Metal Ion Concentration

Exactly, 40 *µ*L of enzyme was incubated with 1 mL of 1 mM solution of the following metal salts such as CaCl_2_, MgCl_2_, MgSO_4_, HgCl_2_, FeSO_4_, and CuSO_4_ and metal chelators such as EDTA for 1 h at optimized temperature (70°C) and pH (9.0 for rice bran and 7.0 for wheat bran). The contents of the flasks were centrifuged at 10000 rpm for 10 min at 4°C and the supernatant was used to assay the enzyme activity at 420 nm.

### 2.15. Statistical Analysis

All the experiments were carried out in triplicate and the results were represented as the independent variables.

## 3. Results and Discussion

### 3.1. Substrate Analysis

The compositions of different natural substrates are shown in Table 1. The effect of substrate concentration on laccase production using* B. subtilis* MTCC 2414 revealed that rice bran and wheat bran showed the maximum enzyme activity of 134.8 ± 4.75 U/mL and 117.6 ± 4.23 U/mL, respectively (Table 2).

### 3.2. Optimization of Process Parameters

**Figure 1:**
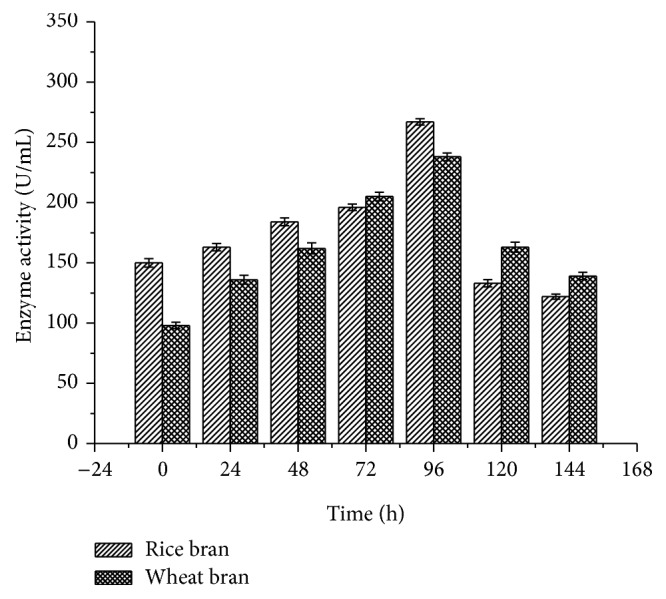
Effect of incubation period on laccase activity.

Among the substrates, a gradual increase in the enzyme activity was noted at the starting time of incubation period and the maximum enzyme activity was attained for rice bran (267 ± 2.64 U/mL) compared to wheat bran (238 ± 3.26 U/mL) at 96 h (Figure 1). But the production was declined at higher incubation time of 144 h.

**Figure 2:**
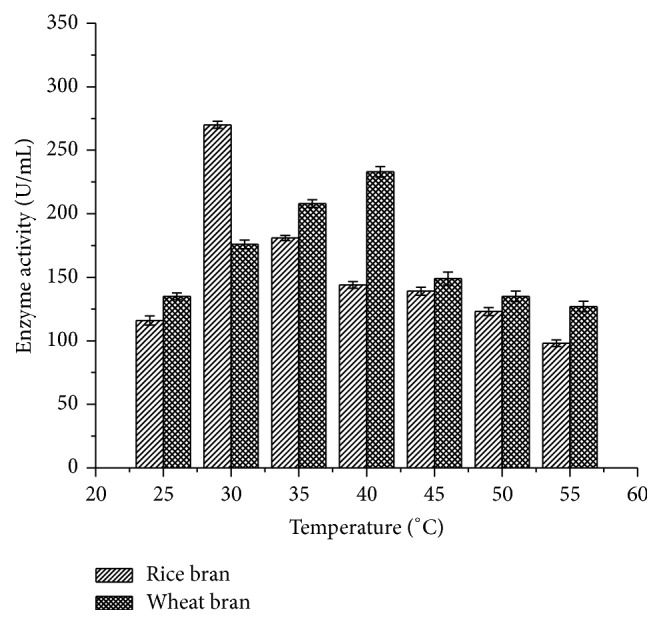
Effect of temperature on laccase activity.

The temperature profile showed differences in the enzyme activity between rice bran and wheat bran. The maximum laccase activity was observed at 30°C (270 ± 2.78 U/mL) for rice bran when compared to wheat bran and 40°C (233 ± 4.09 U/mL) at 96 h of incubation (Figure 2).

**Figure 3:**
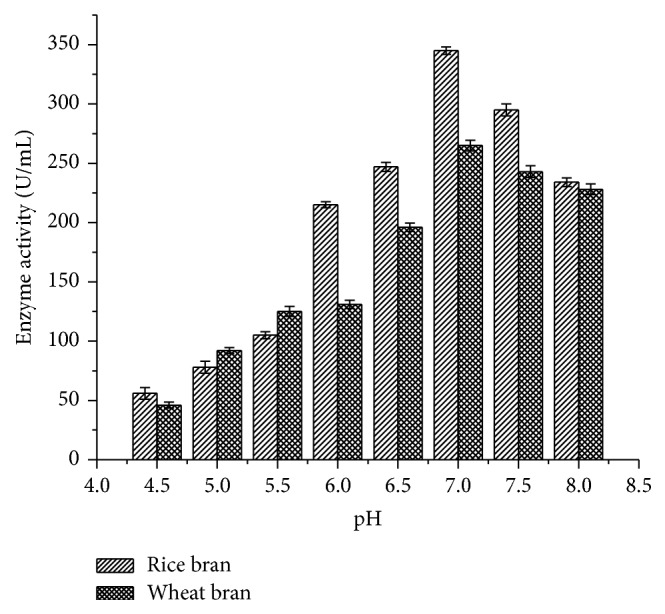
Effect of pH on laccase activity.

The enzyme activity showed a steady state of increase in their activity from pH 4.5 to 6.5, where the maximum activity was attained for rice bran (345 ± 3.14 U/mL) followed by wheat bran (265 ± 4.44 U/mL) at pH 7.0 (Figure 3).

**Figure 4:**
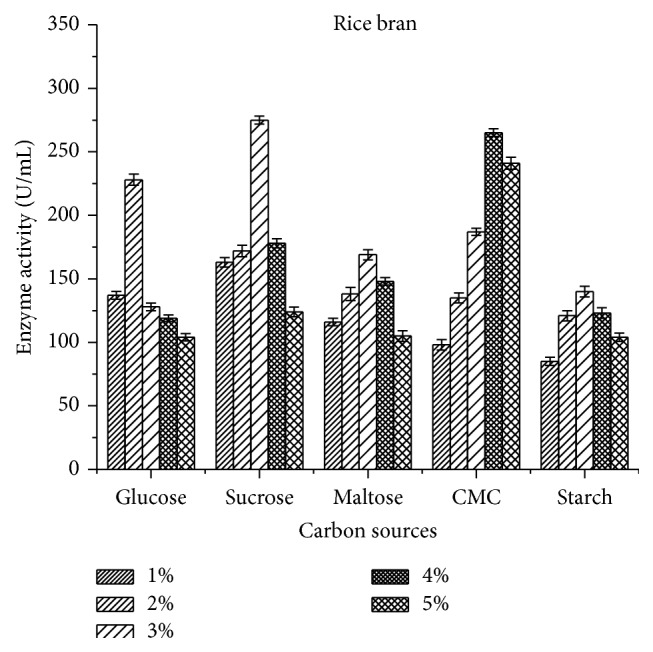
Effect of carbon sources on laccase activity using rice bran as a substrate.

**Figure 5:**
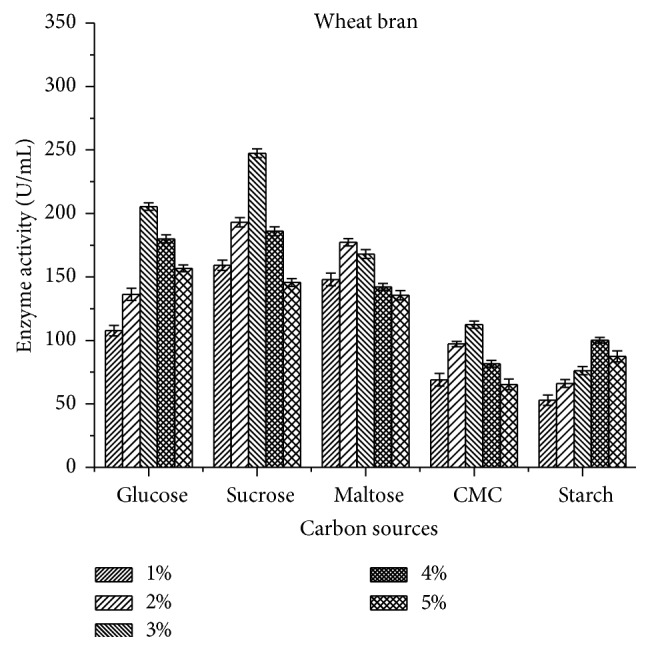
Effect of carbon sources on laccase activity using wheat bran as a substrate.

The present study showed that supplementation of production media with external carbon sources had effect on laccase activity by* B. subtilis* MTCC 2414. On analyzing the results, it was clear that the microorganism necessitates an optimum level of carbon in order to produce enzymes because they also act as a limiting factor. Among the selected carbon sources, 3% sucrose showed the maximum enzyme activity of 275 ± 3.11 U/mL (Figure 4) for rice bran when compared to wheat bran 247.4 ± 3.51 U/mL (Figure 5). However, the minimum activity was observed against starch for both substrates.

**Figure 6:**
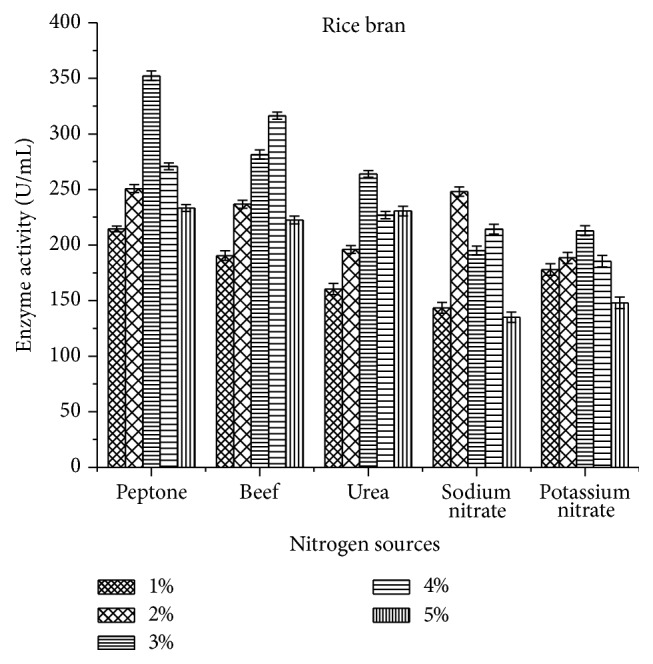
Effect of nitrogen sources on laccase activity using rice bran as a substrate.

**Figure 7:**
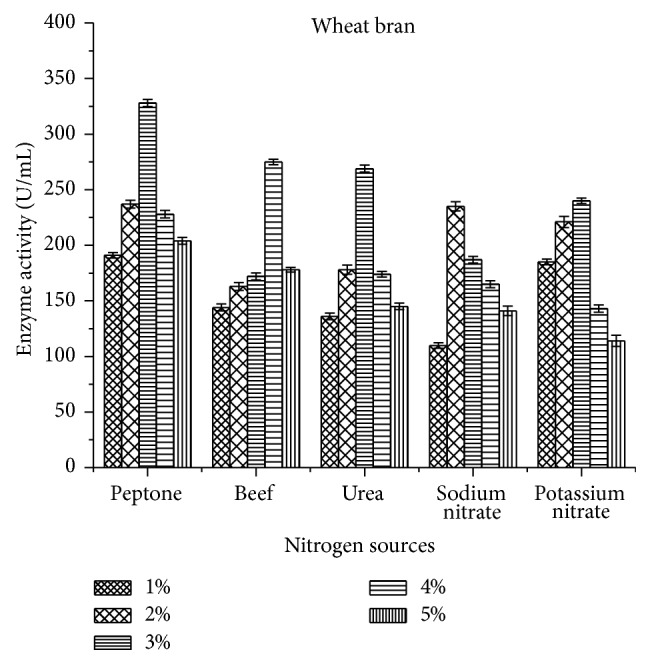
Effect of nitrogen sources on laccase activity using wheat bran as a substrate.

Among the nitrogen sources, organic nitrogen sources showed increased production when compared to inorganic nitrogen sources. In this study, 3% peptone exhibited higher enzyme activity for rice bran (352.2 ± 4.32 U/mL) when compared to wheat bran (328 ± 3.33 U/mL) (Figures 6 and 7). But less comparatively, inorganic nitrogen sources such as sodium nitrate and potassium nitrate had significant role in the laccase production.

### 3.3. Partial Purification of Laccase

During the first purification step, the crude sample obtained after production process was subjected to 80% saturation of ammonium sulphate precipitation where salts were eluted completely. The laccase activity was assayed and exhibited maximum activity of 155 ± 8.03 U/mL for rice bran and 104.15 ± 11.82 for wheat bran. After dialysis, it was found to be 265 ± 8.45 U/mL for rice bran and 232.7 ± 9.25 for wheat bran, respectively. A total of 18.7 ± 4.22 and 15.25 ± 4.41 mg/mL of protein was estimated from partially purified laccase from rice bran and wheat bran, respectively.

**Figure 8:**
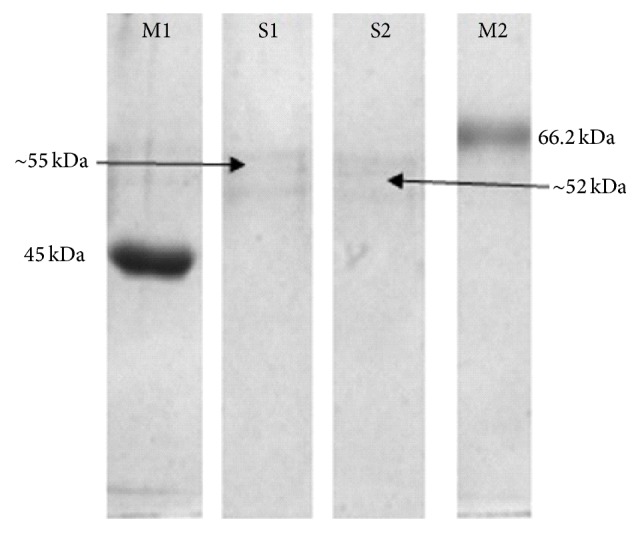
SDS-PAGE of partially purified laccase. M1: bovine serum albumin marker, S1: laccase from* Bacillus subtilis* MTCC 2414 using wheat bran, S2: laccase from* Bacillus subtilis* MTCC 2414 using rice bran, and M2: oval albumin marker.

The molecular weights of partially purified laccase from* B. subtilis* MTCC 2414 using rice bran and wheat bran were determined using SDS-PAGE and they were found to be approximately 52 kDa and 55 kDa for rice bran and wheat bran, respectively (Figure 8).

### 3.4. Enzyme Characterization

#### 3.4.1. Temperature Profile

**Figure 9:**
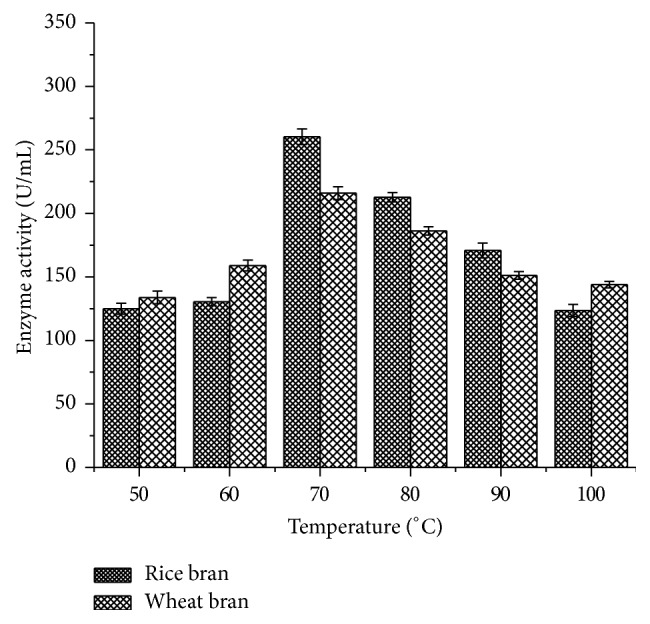
Temperature profile of laccase activity.

The activity of partially purified laccase was examined at different temperatures (Figure 9), and the maximum laccase activity was found to be 260.3 ± 6.15 U/mL for rice bran and 216 ± 5 U/mL for wheat bran at 70°C. However, laccase activity was inactive at higher temperature of 100°C. Major differences in activity were noted between substrates.

#### 3.4.2. pH Profile

**Figure 10:**
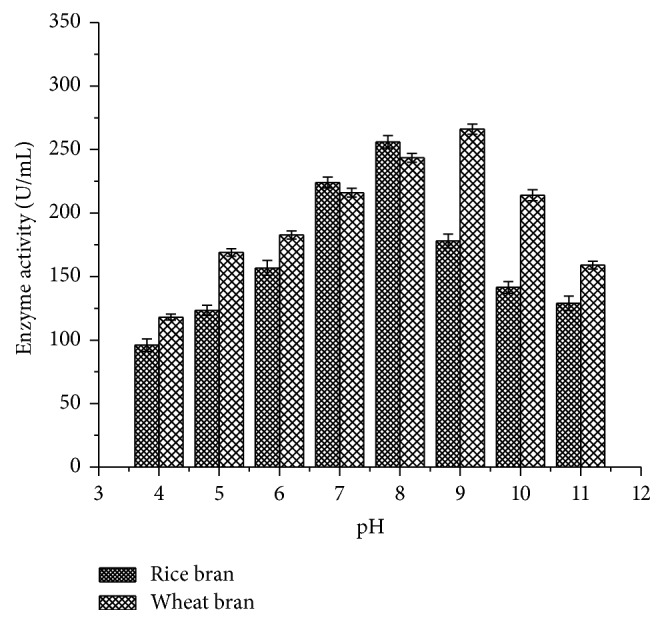
pH profile of laccase activity.

Maximum laccase activity was observed at pH 9.0 for rice bran (266 ± 4.02 U/mL) and pH 7.0 for wheat bran (256 ± 4.94 U/mL) after a period of 3 h (Figure 10). The laccase showed minimal activity in acidic range over alkaline pH range.

#### 3.4.3. Effect of Metal Ions

**Figure 11:**
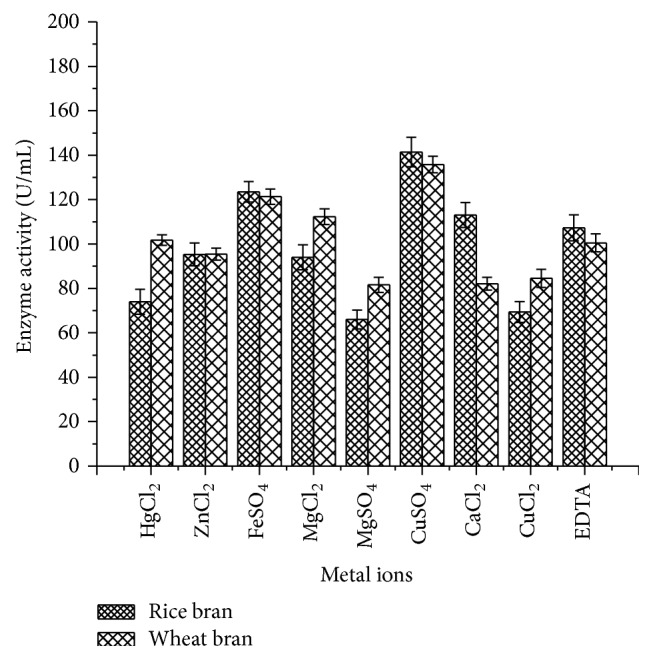
Effect of metal ions on laccase activity.

Metal ion CuSO_4_ enhanced maximum laccase activity of 141.4 ± 6.64 U/mL for rice bran and 135.8 ± 3.65 U/mL for wheat bran followed by FeSO_4_ (123.5 ± 4.59 U/mL for rice bran and 121.3 ± 3.47 U/mL for wheat bran). But, MgSO_4_, CuCl_2_, and HgCl_2_ were found to inhibit the laccase activity to a greater extent. In general, CuSO_4_ was reported to be the inducer for the maximum yield of laccase activity (Figure 11).

The present study has effectively utilized the inexpensive and easily available natural substrates for higher yield of laccase under optimized fermentation conditions. From the selected substrates (Table 2), the maximum enzyme activity (134.8 ± 4.75 U/mL) was observed against rice bran. It can be suggested that laccase utilize xylan, cellulose, and mannan in rice bran as carbon sources during the initial growth phase [18]. The main component of rice bran was expected to be arabinoxylan as most sugars found are xylose and arabinose, and when the levels of those carbon sources decrease, laccase synthesis was induced by lignin and phenolic compounds present in rice bran, leading to an increase in laccase production [19]. Moreover, the nutritional requirement of the organisms is the main factor that determines the suitability of a particular substrate.

*B. subtilis* MTCC 2414 laccase showed maximum activity (267 ± 2.64 U/mL) at 96 h using rice bran as a substrate which was comparatively higher than that of* Schizophyllum commune* laccase (65.5 ± 2.52 U/mL) using rice straw as a substrate [20]. Moreover, laccase produced from* Pycnoporus sanguineus* and* Ganoderma sp*. showed maximum enzyme activity on the 10th and 6th day of fermentation using wheat bran as a substrate [21, 22]. do Valle et al. [23] produced laccase from* Agaricus blazei* using sugarcane molasses and exhibited 140.5 U/mL on the 8th day. This clearly revealed that fungal laccases require longer incubation period than bacterial strains. Longer incubation period is a key factor why bacterial laccases have gained much importance in biotechnological applications. The optimal temperature of laccase differs greatly from one strain to another. Maximum laccase activity (270 ± 2.78 U/mL) of* B. subtilis* MTCC 2414 was recorded at 30°C for rice bran and 40°C (233 ± 4.09 U/mL) for wheat bran. Hullo et al. [24] and Nagai et al. [25] reported that laccase from CotA of* B. subtilis* and* Lentinula edodes* exhibited maximum activity at 40°C. The results of the study are in accordance with recent reports which confirmed that laccases from* P. putida* are highly stable between 30 and 50°C [26].

pH strongly affects the enzymatic reactions and is sensitive to hydrogen ion concentration present in the medium across the cell membrane. Murugesan et al. [27] reported that pH 7.0 was the best for optimal growth of laccase from* T. modesta* and* R. praticola* which is similar to the current study in which maximum enzyme activity of 345 ± 3.14 U/mL was observed at pH 7.0 using rice bran as substrates. An excess of sucrose or glucose in the cultivation media can reduce the production of laccase, as these components allow constitutive production of the enzyme [28]. Among the carbon sources tested, 3% sucrose was found to exhibit maximum enzymatic activity (275 ± 3.11 U/mL) in rice bran followed by 3% carboxy methyl cellulose (112.5 ± 2.89 U/mL) and 2% glucose (228 ± 4.44 U/mL). But Kapdan et al. [29] and Galhaup et al. [30] reported that glucose resulted in improved enzyme production. In another study reported by Fernaud et al. [31], starch-free wheat bran itself was used as a carbon source for high yield of laccase which is in accordance with the current study as starch exhibited lesser laccase activity (100.2 ± 2.5 U/mL).

Among the tested nitrogen sources, 3% peptone (352.2 ± 4.32 U/mL) and 4% beef extract (316.4 ± 3.08) in rice bran resulted in higher laccase production. This was well supported by Morozova et al. [32] where* T. villosa* laccase showed increased production using peptone. De Souza and Peralta [33] reported that organic nitrogen sources enhance laccase production compared to inorganic nitrogen sources and also it may vary with the source of organism [34]. Even in the present study, organic nitrogen sources exhibited maximum activity (352.2 ± 4.32 U/mL) compared to inorganic sources (212.9 ± 4.56 U/mL).

The molecular mass of purified laccase from* P. pulmonarius* was 46 kDa [35] and laccase from* Pleurotus* sp. was 40 kDa. Similarly,* S. psammoticus* laccase had molecular mass of 43 kDa [11] and laccases of 50 kDa from melanogenic bacterium* Bacillus* HR03 [36] were reported in previous studies. However, the molecular masses of laccase from* B. subtilis* MTCC 2414 using rice bran and wheat bran were found to be 52 and 55 kDa which are in consistent with the majority of the previously published reports.

Enzyme activity was observed against different temperatures to determine the thermal stability. The maximum enzyme activity was observed at 70°C (260.3 ± 6.15 U/mL) using rice bran as a substrate which was well supported by Sonica et al. [37] where they have reported comparable activity at 70°C by* B. tequilensis* using softwood pulp as a substrate. Moreover, previous studies have shown maximum laccase activity between 40 and 60°C [38, 39]. The pH profile showed higher laccase activity at 9.0 (266 ± 4.02 U/mL) which is in accordance with Sonica et al. [37]. Kuddus et al. [26] reported that* P. putida* laccase exhibited maximum activity at pH 9 (34.45 U/mL) which is also in accordance with the present study results. Metal ions play a significant role in the laccase production as it induces and suppresses the activity. As laccase is a copper oxidase, CuSO_4_ plays an important role in medium and is also used as a sole inducer [40]. The present study results showed that CuSO_4_ enhanced the laccase activity from* B. subtilis* MTCC 2414 using rice bran (141.4 ± 6.64 U/mL) as a substrate which was consistent with the studies carried out by Telke et al. [38] where* Pseudomonas* sp. LBC1 laccase showed increased activity by the addition of CuSO_4_.

## 4. Conclusion

In the present study, we partially purified laccase from* B. subtilis* MTCC 2414 using both rice bran and wheat bran as a solid substrate. The results clearly proved that rice bran is the potential substrate for higher yield of laccase production compared to wheat bran. The maximum laccase activity and stability were found at 70°C and pH 9.0. In future, the production will be optimized further for enhanced thermostability of laccase which can be used for industrial applications.

## Figures and Tables

**Table 1:** Composition analysis of selected natural substrates.

Natural substrates	Composition analysis (%)					
	NDF	Hemicellulose	Lignin	Cellulose	ADF	Ash
Saw dust	35.8	10.2	11.76	22.31	25.6	3.46
Rice bran	61.12	15.32	16.21	35.4	45.8	5.64
Wheat bran	49.39	13.29	10.64	27.21	36.1	4.28
Banana peel	40.37	12.17	8.21	25.8	28.2	5.31

{NDF: neutral detergent fiber; ADF: acid detergent fiber}.

**Table 2:** Effect of substrate concentration on laccase production.

Natural substrates	Enzyme activity (U/mL)
Saw dust	71.2 ± 3.82
Wheat bran	117.6 ± 4.23
Rice bran	134.8 ± 4.75
Banana peel	75.6 ± 3.61
